# Single nucleotide polymorphism rs696 in miR449a binding site of NFKBIA gene is correlated with risk of colorectal cancer 

**Published:** 2018

**Authors:** Miganoosh Simonian, Meysam Mosallayi, Maryam Miraghajani, Awat Feizi, Sharifeh Khosravi, Ahmad Reza Salehi, Deniz Mortazavi, Farideh Saberi, Rasoul Salehi

**Affiliations:** 1 *Department of Genetics and Molecular Biology, School of Medicine, Isfahan University of Medical Sciences, Isfahan, Iran*; 2 *Cancer Research Center, Shahid Beheshti University of Medical Sciences, Tehran, Iran.*; 3 *Department of Biostatistics and Epidemiology, School of Health, Isfahan University of Medical Sciences, Isfahan, Iran.*; 4 *Acquired Immunodeficiency Research Center, Isfahan University of Medical Sciences, Isfahan, Iran*; 5 *Gerfa Namayesh Azmayesh (GENAZMA) Science & Research Institute, Isfahan, Iran *

**Keywords:** NFKBIA gene, microRNA, single-nucleotide polymorphism, colorectal Cancer

## Abstract

**Aim:**

In present study we have elucidated the role of 2758 A>G (rs696), in the recognition site of miR449a in the 3′ UTR of NFKB inhibitor alpha (NFKBIA) gene, in development of sporadic colorectal cancer.

**Background:**

Colorectal cancer (CRC) is rated as second cause of cancer death. Genetic determinants are considered as driving forces in development of sporadic CRC. Single nucleotide polymorphisms (SNPs), are attributed as the main genetic factor in cancers susceptibility. MicroRNAs, are key players in post-translational gene regulation by binding to their specific recognition sequences located at 3' untranslated region (UTR) of mRNAs.

**Methods:**

A case–control study using 143 CRC patients and 137 noncancerous counterparts were undertaken in order to determine rs696 genotypes using polymerase chain reaction– restriction fragment length polymorphism (PCR–RFLP) method.

**Results:**

There was a significant difference for the genotype frequencies of rs696 between patients and controls. The frequencies of GG, AG, AA genotypes in the control group were 38.7, 45.3, and 16.1 %, respectively, and the genotype frequencies in case group were 19.6, 40.6, and 39.9 %, respectively.

**Conclusion:**

Our results suggest significant correlation between rs696 polymorphism and colorectal cancer risk

## Introduction

 Globally, more than one million people experience CRC every year, and it has become the third and second most common malignancy in men and women respectively (1, 2). Despite this fact that Iran is still a low risk country for CRC, based on the World Health Organization (WHO) reports, its incidence rate currently showing rapid increase perhaps due to the Westernization of lifestyle in Asia countries, including Iran (3, 4). Approximately 80% of all CRC cases are sporadic, indicating that besides genetic basis, environmental and lifestyle factors also play important roles. Alcohol, tobacco, type of diets, physical activity, nonsteroidal anti-inflammatory drugs (NSAIDs) usage and inflammatory bowel disease (IBD) are important risk factors for CRC development (5-7). There is a growing body of evidence that inflammatory microenvironment is an essential component of all cancers, especially colon tumors (8). Chronic inflammation is likely to be implicated in various forms of sporadic as well as heritable colon cancer (8). As a crucial inflammatory element nuclear factor kappa B (NFKB), could have an oncogenic role by promoting the growth and survival of many solid malignancies (9) . NFKB seems to be activated in wide fraction of tumors such as breast, colon, prostate and stomach and implicate to tumor progression and angiogenesis (10-13). Meanwhile IKBα, the inhibitor of NFKB, has been reported to be inactive or downregulated during various NFKB activation processes (14). NFKB/IkBa complex is a central hub in networked cellular transcriptional responses; with wide impact on expression of different genes. Its dysregulation contributes to important cellular phenomenon like angiogenesis, cell proliferation, invasion and anti-apoptosis processes favoring oncogenesis (15).

MicroRNAs (miRNAs), small noncoding RNA molecules with ≈22 nucleotides, have attracted great attentions because of their principle role in various aspects of cellular biology such as cell differentiation, proliferation, cell cycle progression and apoptosis (16, 17). miRNAs could affect the level of mRNA expression through hybridization to complementary sequences in the 3' untranslated region (UTR) of the target gene transcripts (18). Recent studies have indicated that single nucleotide polymorphisms (SNPs) in miRNA binding sites could modulate miRNA-mRNA interactions and change gene expression drastically by either creating new or deleting existing miRNAs binding sites (19). 

In silico analyses it was indicated that *NFKBIA* 2758 A>G (rs696) variant located at recognition site of miR449a (20). Moreover Song *et al.* (20) ex vivo luciferase data manifested that 2758 A allele thermodynamically favor stronger binding capacity of miR-449a with 3’UTR of *NFKBIA*, which result in decreased expression of *NFKBIA* and in turn constitutive activity of *NFKB*. Therefore, individuals carrying AA genotype at rs696 lesser amount of NFKB inhibitor and unchecked NFKB remain with prolonged activity which is in favor of infectious and inflammatory diseases as well as tumorigenesis (21). Environmental factors like NSAID use, smoking, physical activity in relation with rs696 genotypes and their influence on colorectal cancer susceptibility was also worked out. Given that *NFKBIA* polymorphisms have not studied yet in Iranian population. The aim of our study was to examine the risk associated with variants of rs696 located in 3’ UTR of *NFKBIA *gene among a group of Iranian patients diagnosed with sporadic CRC. 

## Methods

**Study population and sample preparation**

A total of 143 patients with histologically confirmed colorectal cancer and no familial history of related cancers were enrolled for this study. Patients were recruited from colonoscopy units of Alzahra and Syedolshohada hospitals between June 23, 2013 and July 15, 2015. Hundred and thirty seven individuals with no evidence of colonoscopy signs of CRC were randomly selected from the same residential areas, during the same period of time. This study was approved by the university ethics committee and all participants provided the written informed consent. The study participants were interviewed and data on gender, age, smoking status, NSAID usage, physical activity and other factors including family history of cancers were obtained using a structured questionnaire. All mandatory applicable laboratory health and safety procedures related to this research have been fully observed and practiced.

**DNA extraction and NFKBIA genotyping**

**Figure 1:**
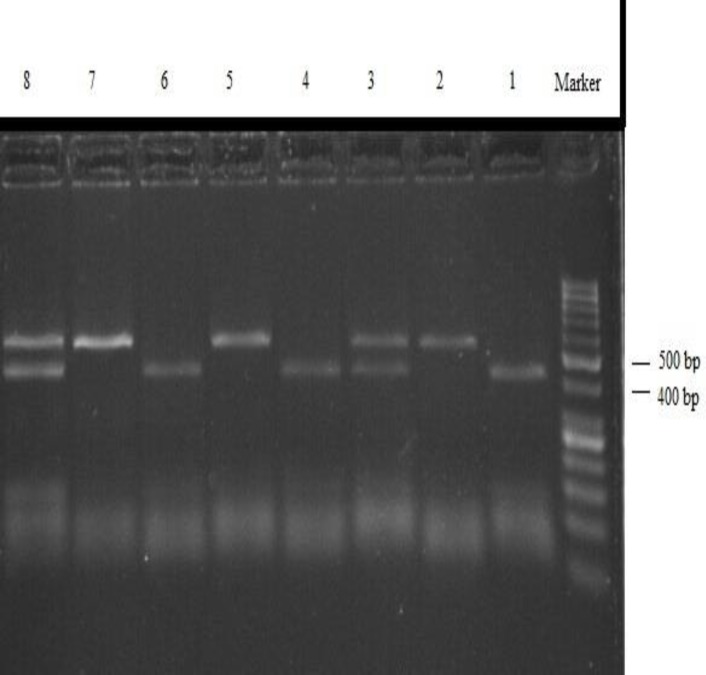
Agarose gel electrophoresis analysis of restriction fragment length polymorphism-polymerase chain reaction products. Lines 1, 4 and 6 are GG genotype, 2, 5 and 7 are AA genotype, and 3 and 8 are AG genotype

Genomic DNA was extracted from 5ml EDTA-anticoagulated peripheral blood samples by Prime Prep Genomic DNA Isolation Kit (GeNetBio, Korea). DNA was stored at -20^o^C until analyzed. The quality and quantity of the extracted DNA was assessed by agarose gel electrophoresis and spectroscopy. The polymerase chain reaction fragment length polymorphism (PCR-RFLP) method was used to determine rs696 genotypes. PCR was performed in a total 25µl volume containing, 2.5 µl 10x PCR buffer, 1.5 mM MgCl2, 200 μM dNTPs, 0.2μM each primer, 100 ng of genomic DNA, and 1 U of Taq DNA polymerase. Thermal cycling profile adjusted at 94^◦ ^C for 5 min followed by 34 cycles of 94^◦ ^C, 64^◦ ^C and 72^◦ ^C all for 30 seconds with a final extension step of 72^◦ ^C for 5 min. Primer sequences, reaction conditions, and restriction enzyme used are given in Table 1. The PCR products were digested with *HaeIII *restriction endonuclease enzyme overnight at 37^◦ ^C. Subsequently restriction enzyme was inactivated at 80^◦ ^C for 20 minutes and then analyzed on 2% agarose gel. Fragment sizes of 535 and 410 bp were indicative of AG heterozygous genotype; a single 410 bp fragment displayed the presence of GG homozygous and one fragment of 535 bp indicated AA genotype (Figure 1). To confirm RFLP genotyping results some of the samples were randomly selected and subjected to direct sequencing.

**Table 1:** Primer sequence and characteristic of PCR product and digestion reaction

SNP ID	Primer sequence	Primer (bp)	Restriction enzyme (Ta˚C)	RFLP fragment size (bp)
Rs696	F:CGCAAAGGGGCTGAAAGAACAT R:TGTGAGAAGCTGCTGGGAGTTA	535	HaeIII(37˚C)	GG:410+ 125+84 AA: 535+84 AG: 535 + 410 +125+84

**Table 2:** Baseline characteristics of CRC patients and controls in study

	Controls (N: 137)	Cases (N: 143)	P value
**Age** (mean ± SD)	55.77±13.25	56.60±10.33	0.56
**gender**			
male		75(52.4%)	0.02*
female		68(47.6%)	
**smoking**			
Yes		28(19.6%)	0.34
No	116(84.7%)	115(80.4)	
**NSAIDs**			
irregular	101(73.7%)	125(87.4%)	0.004*
regular	36(26.3%)	18(12.6%)	
**Physical activity **			
low	82(59.9%)	108(75.5%)	0.007*
moderate	34(24.8%)	27(18.9%)	
high	21(15.3%)	8(5.6%)	
**BMI** (mean ± SD) kg/m2	25.82±4.03	27.33±7.77	0.10

* P value < 0.05

**Table 3:** Association between genotypes and allele frequency with CRC risk

	Case No (%)	Control No (%)	*P* value	OR (95 %CI)
**Genotype frequency**				
GG	28(19.6%)	53(38.7%)	<0.001*	3.46 (1.96-6.10)
AG	58(40.6%)	62(45.3%)		
AA	57(39.9%)	22(16.1%)		
**Allele frequency**				
G	114 (39.9%)	168(61.3%)	<0.001*	2.39(1.70-3.35)
A	172(60.1%)	106(38.7%)		

* P value < 0.05

**Table 4:** Association of NSAIDs consumption with genotype

Group	Genotype		*P* value	OR (95 %CI)
	GG+AG	AA		
**regular NSAIDs Consuming**				
Case	7(19.4%)	11(61.1%)	0.002*	6.51 (1.85-22.87)
Control	29(80.6%)	7(38.9%)		

* P value < 0.05

**Statistical analyses**

All data analyses were carried out using SPSS 22.0 statistical software. The t- test and the Chi-squared (χ^2^) test were used to compare the differences of demographic characteristics distributions between CRC patients and the control group. Hardy–Weinberg equilibrium was tested among cases and controls using Pearson’s Chi square test. Genotype and allele frequency differences between cases and controls were evaluated using logistic regression analysis, with odds ratios (OR) and 95% confidence intervals (95% CI). *P *value < 0.05 was considered statistically significant. 

## Results

Our study population consisted of 143 CRC patients (75 males and 68 females) and 137 CRC free individuals (54 males and 83 females). The distributions of selected characteristics of the cases and controls are presented in Table 2. There were no statistically significant differences between patients and controls in terms of BMI and smoking status (*P *= 0.10 and* P *= 0.34 respectively). However, we found a significant difference in physical activity (*P* = 0.007) between case and control groups. Also individuals in control group were more aspirin or NSAID users compared with sporadic CRC cases (*P* < 0.004). The case group had a higher proportion of male subjects (52.4% vs 39.4%). 

Genotyping data were in Hardy–Weinberg equilibrium for this SNP in the tested groups. There was a significant difference for the genotype frequencies of rs696 between patients and controls. The frequencies of GG, AG, AA genotypes in the control group were 38.7, 45.3, and 16.1 %, respectively, and the genotype frequencies in case group were 19.6, 40.6, and 39.9 %, respectively. This result demonstrated the significant association of AA genotype with risk of CRC in our study population (adjusted OR= 3.46; 95%CI [1.96–6.10];* P *< 0.001). In allele distribution analysis we found increased level of allele A in cases, compared with controls. This allele was associated with an increased risk of CRC (adjusted OR= 2.39; CI [1.70–3.35]; *P *< 0.001). The distributions of genotype and allele frequency are shown in Table 3.

As mentioned above we also found that our case group consumed less aspirin or NSAID compared to control group (73.7 vs. 87.4 %, *P* = 0.004). The analysis for correlation between *NFKBIA* genotypes and NSAIDs consumption demonstrated that in drug user patients, AA genotype was more frequent vs. AG, GG genotype (61.1%vs. 19.4%, *P* = 0.002). While in control group, the situation was reverse (38.9% vs. 80.6%). Data are shown in Table 4.

## Discussion

In the present study, we found that the genetic variant, rs696 A>G of the NFKBIA gene significantly correlates with colon cancer in Iranian population. Analysis indicated that homozygous AA genotype (adjusted OR= 3.46; 95%CI [1.96–6.10]) was more frequent in CRC patients. Also individuals possessing allele A were more frequently affected with CRC than those with G allele (adjusted OR= 2.39; CI [1.70–3.35]). 

In a study conducted in China AA genotype of rs696 polymorphism found to be correlated with elevated risk of nasopharyngeal carcinoma (22). Such association has also been confirmed with Behçet’s disease and lung cancer in Turkish and Chinese populations respectively (23, 24). AA variant involvement in increased risk of aforementioned malignancies must be due to the downregulation of IkBa expression because of the modulation of miR-449a binding site on 3’UTR of the gene. On the other hand, the correlation of GG genotype with an increased risk for extensive colitis in Hungarian patients is inconsistent with the above mentioned findings. Also subjects with GG genotype determined to be at twofold higher risk of cervical cancer as compare to controls (25). Curran *et al*. (26) could not find correlation of this SNP with susceptibility to sporadic breast cancer in Caucasian population. Moreover, Geo *et al.* (27) indicated that in Chinese population AG genotype was more in CRC patient with ≥50 years, while GG genotype considered as prognostic factor in Swedish CRC patients. Taken together, these contradictory results suggest that the correlation of this polymorphism with cancers might vary among various populations due to differences in ethnic background and different types of cancers and highlight the importance of population based studies more than before.

To examine the interaction between the NFKBIA genotypes and NSAID use, we assessed the effect of NSAID use stratified by NFKBIA genotypes in drug user subgroup. The result showed that patients with AA genotype of this polymorphism appeared to have less benefit of NSAID use; it is plausible that NSAID use could affect by NFKBIA genotypes. Without doubt, agents such as NSAIDS that inhibit inflammatory events can reduce the risk of CRC. Based on the available reports 50% diminution in the incidence of colorectal cancer attributed to the regular aspirin users (28, 29). But our data may lead to this conclusion that all individuals consuming NSAID would not get equal protection, their genotype is an important determinant in drug utility and protection against CRC development. In order to make a conclusive exploration of these potential interactive effects, larger well-powered studies are needed.

We believe this work would further justify the role of NFKB1/NFKBIA axis in colon cancer susceptibility. Further evaluation of the role of rs696 in NFKBIA gene in other population with an expanded population size would help to reach a definite conclusion regarding the role of this polymorphism and may prove its utility as a CRC screening biomarker.
